# From Trap to Nursery. Mitigating the Impact of an Artisanal Fishery on Cuttlefish Offspring

**DOI:** 10.1371/journal.pone.0090542

**Published:** 2014-02-28

**Authors:** Valentina Melli, Emilio Riginella, Marco Nalon, Carlotta Mazzoldi

**Affiliations:** Department of Biology, University of Padua, Padua, Italy; University of Bologna, Italy

## Abstract

**Background:**

Overexploitation and the impact of several types of human activities have caused declines of marine resources. The direct and active involvement of fishermen in the management of marine resources is effective not only for resource conservation, but also for changing fishermen’s attitudes. In this study, we proposed and tested the efficacy and suitability of a measure for mitigating the impact of a trap fishery on cuttlefish eggs in the North Adriatic Sea. This measure directly involves fishermen in promoting the conservation of the early, and more vulnerable, stages of the cuttlefish life cycle.

**Methodology/Principal findings:**

Through surveys on fishing boats and interviews with fishermen, we found that traps placed in coastal areas during the cuttlefish breeding season have a high impact on cuttlefish eggs, with over 3 million eggs likely being destroyed by 3750 traps of 15 fishermen in less than 3 miles of coast. The use of removable ropes attached inside traps as an additional substrate for egg deposition allowed the recovery of 23.7% of the eggs deposited on the traps on average, without affecting the catch rate of adults. Experiments examining hatching success in the field highlighted the need for a careful choice of hatching sites to maximise the efficacy of the mitigation measure.

**Conclusions/Significance:**

The proposed mitigation measure reduced the impact of fishing on cuttlefish eggs, with no significant effect on the commercial catch. Fishermen showed a positive attitude towards the application of this measure, which is inexpensive and easy to employ. The direct involvement of fishermen in the management of this resource and the maintenance of traditional fishing methods are a novel aspect of the proposed measure and represent the basis for its success.

## Introduction

Marine resources are directly and indirectly impacted by human activities such as fishing, pollution, transformation of marine habitats and anthropogenic climate change [1], [2], [3]. These different impacts often act synergistically on marine resources. Habitat loss and degradation are particularly severe in coastal areas, which have historically been subjected to the strongest human impacts [4], [5], [6]. Coastal areas host high biodiversity and provide sensitive habitat, such as seagrass meadows, which are essential for the reproduction and/or growth of a number of marine species, including commercially exploited organisms [7], [8], [9].

Fishing activities have direct and indirect impacts on both target and non-target species, marine communities and natural habitats [10], [11]. Reduction of abundances and changes in growth, production and recruitment are the most common effects observed on target and by-catch species [10]. Such effects may be a direct outcome of the removal of individuals, the end result of interference of fishing activities associated with breeding events [12] and/or an indirect consequence of the destruction of essential habitat and changes in trophic webs and community structures [10]. Fishing activities are characterised by different selectivities and impacts [10], [11]. Artisanal fisheries generally produce less discard and have a lower impact than trawling, particularly bottom trawling [10], [11]. For instance, in the Mediterranean Sea, it has been estimated that artisanal fisheries usually discard less than 15% of their catch, while trawls discard 20 to 70% of their catch [11]. In the Mediterranean Sea, the artisanal fisheries employ small boats and fish near the coast using different gear types (different types of traps, gill and trammel nets or long-lines) [13], [14]. The different gears are employed to target seasonally different species, often exploiting seasonal variations in species behaviour and/or habitat use [13]. Several measures aimed to reduce or mitigate fishery impact have been developed worldwide. These measures are usually gear and site specific, being the impact of fishery tightly linked to gear design and target species [10], [11]. In order to be employed, the efficacy of these mitigation measures need to be experimentally tested in the real-world condition and the effect on fishermen’s income evaluated [15]. Indeed, while the efficacy of a measure aimed to mitigate fishery impact is a prerequisite to forecast its positive effects on natural stocks, effective mitigation measures that highly reduce catch are less likely to be accepted and adopted by fishermen.

Management measures targeting marine resources have often been unable to prevent stock depletion or effectively promote stock recovery [16]. A co-management approach, with the direct and active involvement of fishermen, is currently recognised as crucial for the success of management actions [16], leading to positive results in different communities, especially for small-scale artisanal fisheries (see, for instance [17], [18], [19]).

The common cuttlefish, *Sepia officinalis* Linnaeus, 1758, occurs in the Eastern Atlantic, from the Baltic and North Seas to South Africa, and in the Mediterranean Sea, where in particular it represents an important and valuable fishery resource [20].

High fishing pressure is generally exerted on spawning adults, taking advantage of spawning migrations to coastal areas, where mating occurs and females attach eggs to substrates such as seagrass, polychaete tubes, ropes and other artificial surfaces [21]. Such migrations produce a high and localised abundance of individuals, easily targeted by fisheries worldwide, particularly by vessels using traps [22], [23], [24], [25], [26]. Regardless of their differences in shape and structure, traps exploit female attraction to deposition substrates and male attraction towards females and are highly selective, almost exclusively capturing mature breeding individuals. Consequently, females often lay eggs on the trap surface. Considering that the development of cuttlefish eggs last from 20 to 50 days [27], traps are often completely covered by cuttlefish eggs after a few weeks of fishing activity [22]. To prevent a reduction of the fishing capacity, eggs are actively removed from the fishing gear with highly destructive devices (pressure washers). Moreover, the cleaning conducted at the end of the fishery season results in the destruction of all the last-laid eggs, whose development would require weeks [22], [28]. In Morbihan Bay, France, it has been estimated that this practice causes the destruction of 18–40 millions of eggs per season [22]. Egg destruction, together with the reduction of coastal seagrass meadows, is likely contributing to declines in the cuttlefish catch recorded by various fisheries in the last decade [20], [28], [29], stressing the importance of developing management actions to preserve this resource. To our knowledge only a single compensatory measure, involving the provisioning of artificial substrates for egg deposition, has been tested previously [22], [28]. Nevertheless, this strategy would not be easily applicable in areas such as the northern Adriatic Sea, where hydraulic dredges are allowed to fish inshore, as the presence of artificial substrates would interfere with this fishery.

The present study, performed in the northern Adriatic Sea, aimed to 1) estimate the annual cuttlefish egg losses associated with fishing traps, in the study area; 2) test the efficacy of a mitigation measure to reduce egg loss; 3) evaluate the consequences of this mitigation measure for fishermen, in terms of the cuttlefish catch.

## Methods

### Ethics Statements

The onboard fishery surveys were authorised by the Coast Guard of Chioggia (Italy) (N° 1758, 9976 and 15438; 05.01_G.M./PESCA), and all cuttlefish and eggs were collected during normal fishery procedures, with no additional experimental catches being performed. The egg hatching experiments were performed either under natural conditions (in the field, close to the deposition site), including only surveys of hatching rates, or in the laboratory, where all of the hatched cuttlefish were immediately collected and released in the field. The Hydrobiological Station “Umberto D’Ancona” where laboratory hatching experiments were performed is permitted to house animals by the Veterinary Service of the local Azienda Socio Sanitaria N. 14 of Chioggia, protocol 353/V 02/04/2001. According to Italian Law DL116/92 and European Directive 2010/63/EU, this study did not require authorisation from the Ministry of Health because no experiments were conducted on the animals, and the hatching experiments did not cause pain, suffering, distress or lasting harm.

### Study Area

**Figure 1:**
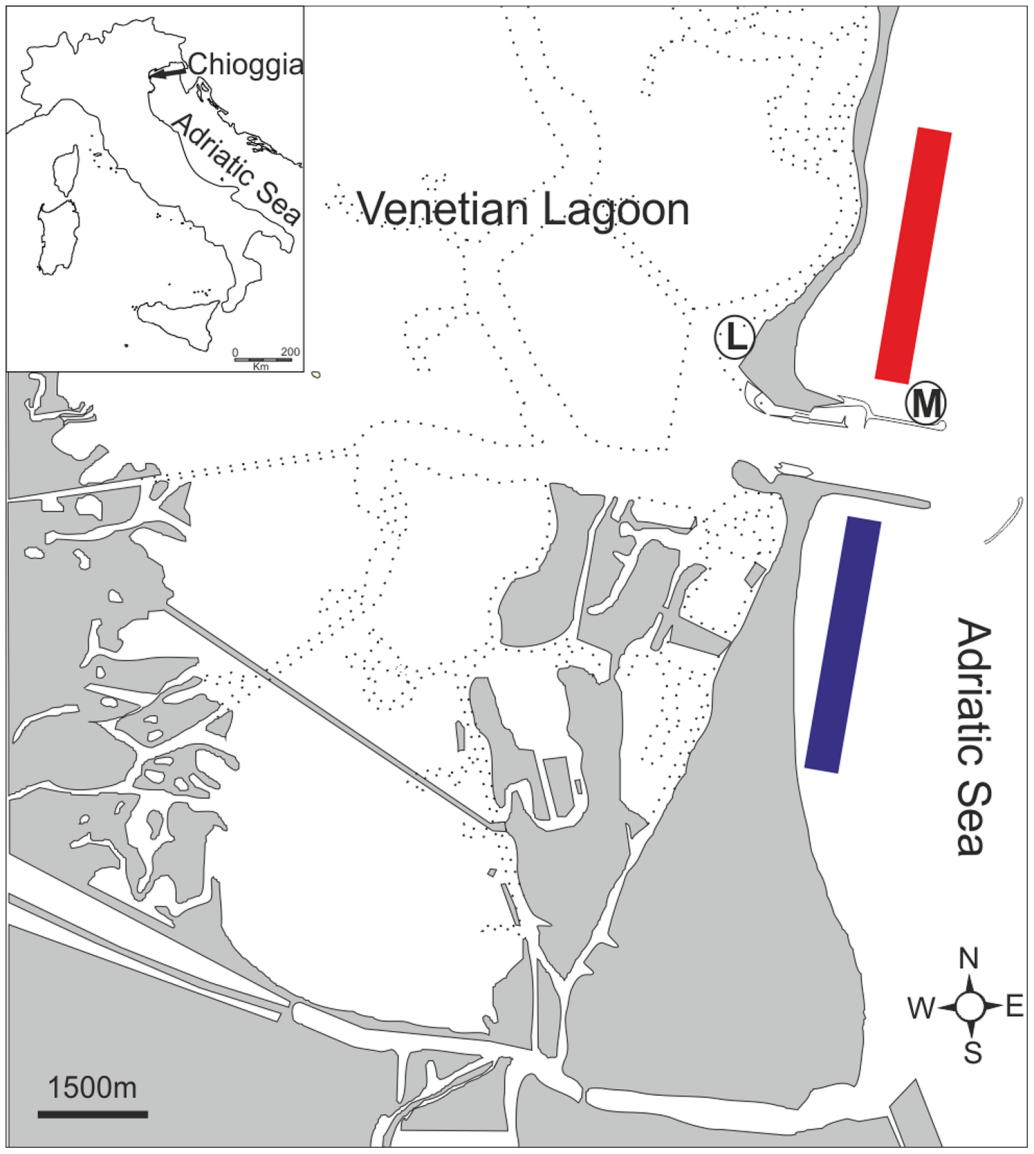
The study site. Red and blue bars indicate the fishery grounds of the two fishing boats involved in the experiments; M and L indicate the marine and the lagoon hatching sites, respectively.

**Figure 2:**
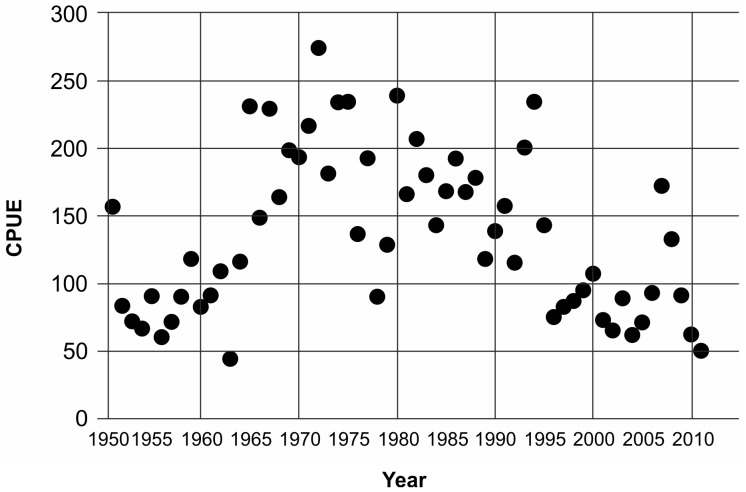
Cuttlefish CPUE in the northern Adriatic Sea. The catch per unit of effort (CPUE) of the fishing fleet of Chioggia (the major fleet in the northern Adriatic Sea) from 1951 to 2011 is reported [29]. The landings are divided by the fishing capacity (total gross tonnage), which is the only available measure of fishing effort [29], [41]. Other cuttlefish species registered in the same category as the common cuttlefish in the time series obtained from the fish market of Chioggia constitute less than 2% of this category on average; therefore, the time series reflects trends of common cuttlefish landings.

The study was conducted in the north-western Adriatic Sea, at two coastal sites, located north and south of one of the channels connecting the Venetian Lagoon to the open sea (Figure 1). The northern Adriatic Sea is a Mediterranean sub-basin with a surface of c. 32000 km^2^, characterised by very shallow depths (up to 100 m, 29 m on average) and by sandy-muddy bottoms on the western side and rocky bottoms on the eastern side [30]. The large nutrient loads carried mainly by northern Italian rivers result in a eutrophic status of this area [31]. Given its high productivity, the northern Adriatic Sea is possibly the most exploited basin of the Mediterranean Sea [32], [33]. Chioggia’s fishing fleet, which is one of the most important in the entire Mediterranean Sea, included, in 2011, 248 fishing vessels equipped with bottom trawling (otter and beam), mid-water trawling, hydraulic dredge, gill and trammel nets as well as traps [29]. The annual cuttlefish landings of Chioggia’s fishing fleet are 784.9±281.4 tons on average, representing 8.8±2.5% of the total landings of the fleet (data from 1997 to 2011; [29]). This resource showed an increase in the catch per unit of effort (CPUE) from 1950s to the beginning of 1980s, which then began to decline (Figure 2; [29]).

Cuttlefish are fished throughout the year using different techniques: in winter, together with other species, they are fished using bottom trawls in the open sea, whereas in spring, when they migrate to the coast and to lagoons for reproduction, cuttlefish are specifically targeted with traps and trammel nets. Before 2010, small otter trawls were allowed by a special authorisation for fishing in coastal waters during the breeding period. Since 1^st^ June, 2010, this fishing method has been banned.

The fishing of cuttlefish using traps is regulated by the Coast Guard of Chioggia at the southern study site and the Coast Guard of Venice at the northern site. Data on the number of licenses allocated are available only for the southern site, where in 2011 15 boats were allowed to employ 250 traps each, operating in assigned areas along the coast.

### Data Collection

**Figure 3:**
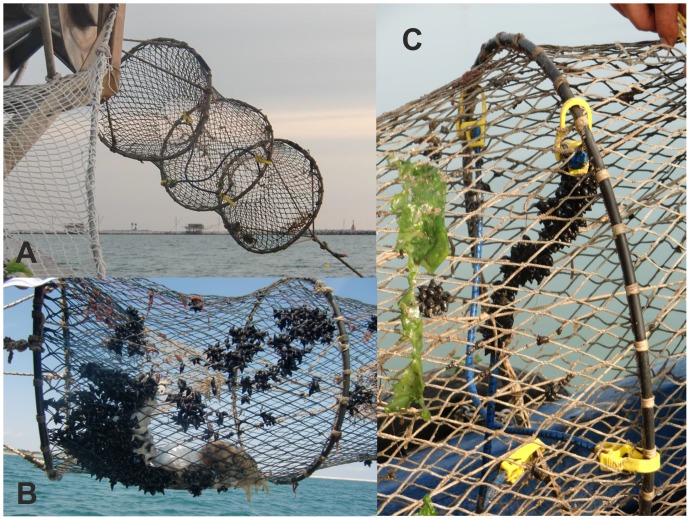
Traps and the mitigation measure. A) trap with ropes; B) trap with cuttlefish and eggs; C) elastic rope inside a trap with cuttlefish eggs.

The work described herein was carried out during two consecutive fishery seasons: 2010 and 2011. Starting from an old practice of fishermen to put inside traps branches of vegetation, especially laurel oaks, to attract inside cuttlefish, we tested a mitigation measure consisted of using ropes hooked inside traps as additional/alternative substrates for egg deposition (Figure 3).

In 2010, a pilot study was carried out to 1) determine where and how to place the ropes inside traps; 2) test whether the ropes were utilised for deposition; 3) verify whether colour influenced female deposition, using blue and yellow elastic ropes [22]; and 4) estimate hatching rates in the laboratory. In 2011, the mitigation measure was tested in terms of 1) its efficacy, estimating the numbers of eggs on ropes relative to the numbers on traps and hatching rates in the field; and 2) the effects of the presence of ropes on the cuttlefish catch. Egg deposition on ropes consisting of different materials (hemp and elastic) was also compared (3).

### Fishery Data

Two fishermen, operating at the northern (Boat 1) and southern (Boat 2) sides of the inlet of Chioggia (Figure 1), were involved in the research. Ropes with a diameter of 8 mm, following Blanc and Daguzan [22], and a length of 40 cm, were hooked transversely on the middle rings of the trap using numbered plastic clips, to allow rapid removal (Figure 3A).

In 2011, at the beginning of the fishery season (March), 30 traps were randomly selected for each fisherman: 10 were equipped with two elastic ropes, 10 with two hemp ropes and 10, randomly chosen in each survey, were used as controls. During the entire fishery season (March–June), we came on board with each fisherman once a week for 7 weeks, for a total of 14 surveys. During each survey, we recorded 1) the number and total weight (to the nearest 5 g, using a dynamometer) of caught cuttlefish; 2) the number of eggs laid on the traps (taking 3 to 5 digital photographs of each trap, followed by analysis with an object counting software -Software “Object counter” developed by A. Sambo, University of Padua - that allowed to manually mark with a spot each egg in the photograph and, keeping tracks of the number of spots, provided the count of the total number of marked objects); and 3) the length of the rope covered by eggs (to the nearest 0.5 cm, using a ruler). Considering that the eggs often hung in aggregates on the ropes, to estimate total egg number on the ropes, the average number of eggs/cm was calculated using 120 direct counts. The reliability of the estimation was then tested on 67 ropes. No significant differences were found between estimates and direct counts (t test for paired data: t_66_ = 1.99, p = 0.610), and the two values were highly correlated (Spearman correlation: r_S_ = 0.92, p<0.0001). During each survey, if eggs covered more than 50% of the rope, the rope was replaced with a new one of the same material; otherwise, the rope was left in the trap. The removed ropes were kept in buckets with seawater (collected directly at the fishing sites) and aerators and brought to the laboratory (first year) or to field sites (second year) to estimate hatching rates. The transport from the fishing sites to the laboratory or field sites lasted on average one hour, and seawater was changed during the trip.

### Hatching Rates

Hatching rates were first estimated in the laboratory to investigate whether the ropes affected egg hatching. At the Hydrobiological Station “Umberto D’Ancona” of Chioggia, ropes with eggs were placed in outdoor tanks provided with continuous seawater flow and aerators. As a control, egg samples were gently removed manually from the traps and placed under the same conditions. Each sample was kept separate, and the eggs were counted. Hatching success was estimated by counting the number of hatchlings. After hatching, the cuttlefish were released.

Hatching rates in the field were estimated at two natural sites, one inside the Venetian Lagoon and one on the coast near the fishing area (Figure 1). The two sites present different characteristics. The lagoon site is sheltered, with a muddy bottom and a depth of 1.5 m, and is not located on a lagoon channel. The marine site is located on a jetty, therefore presenting an artificial hard bottom, with a depth of 4 m, and it is exposed to north-eastern winds. At both locations, exploiting a pre-existing structure, a steel cable was horizontally fixed at a depth that guaranteed total submersion of the eggs (water depths above 0.5 m and 2 m at the lagoon and marine sites, respectively) and prevented the ropes from touching the bottom (water depths below 0.4 m and 1.5 m at the lagoon and marine sites, respectively), thus avoiding damage to the eggs and minimising the risk of egg predation by benthic organisms, where the main predator is the gastropod *Hexaplex trunculus* (V. Melli, personal observation). The ropes were hooked to the cable and loaded with a weight of 200–300 g to increase stability and distributed with a distance of 50 cm between ropes to avoid any contact between them. The ropes were assigned randomly to the hatching areas, with 7 hemp and 13 elastic ropes being allocated to the marine site and 8 hemp and 6 elastic to the lagoon site. Egg development was checked once a week through photographing the entire rope, and the numbers of eggs that hatched, degenerated or were developing were quantified. Hatched eggs were recognised by an envelope containing a hole and that was still attached to the rope; they were discriminated from the predated eggs because the rest of the envelope was completely intact. Eggs were considered degenerated if they became opaque and gelatinous (based on laboratory observations), or if a sessile organism proliferated on them. Developing eggs did not appear opaque and increased in size from week to week [34]. The egg loss due to detachment or, possibly, fish predation, was estimated as the difference between the number of eggs at the time of rope collection and the total number of hatched and degenerated eggs during the entire development period. Abiotic parameters that might potentially influence egg hatching were monitored in both areas. At each control survey, the surface temperature was measured in the field using a thermometer (to the nearest 0.1°C). A water sample was brought to the laboratory to estimate salinity, using a scale of densimeters (Richter and Wiese); pH, with a pH-meter (CRISON) calibrated with NBS buffer; and oxygen contents (mL/L), via Winkler titration.

### Interviews

All 20 fishermen (15 from the Chioggia fleet and 5 from the Venice fleet) operating cuttlefish traps in the study area and 8 fishermen operating within the lagoon were interviewed. They were asked 1) to quantify the coverage of eggs on their traps in the last 5 years as Absent, Scarce (covering less than 1/2 of the trap), Intermediate (approximately 1/2) or High (more than 1/2); 2) whether they usually clean eggs from their traps, indicated as No, Rarely or Yes; 3) the cleaning method adopted, as Nothing, By hand, Pressure washer or Sun drying; 4) for possible alternatives to reduce impacts on eggs; and 5) their opinion regarding the proposed mitigation measure.

### Data Analyses

Data are presented as the mean ± standard deviation. Statistical analyses were performed using STATISTICA 7.1 software, PRIMER 6 and PERMANOVA plus [35]. Parametric or non-parametric analyses were applied according to data distribution and test assumptions.

The overall impact of the traps on cuttlefish eggs in the study area was estimated as the product of the estimated number of eggs per trap at the end of the fishery season and the number of traps employed in the study area (along 2.96 miles of coast). The approximate number of cuttlefish clutches impacted by traps was estimated considering that female cuttlefish lay from 150 to 550 eggs per clutch [27].

To investigate whether the presence of the ropes affected the catch of cuttlefish in the traps, the number and weight of caught cuttlefish (log-transformed) were used to build matrices of Euclidean distances. The matrices were then tested through Permutational Multivariate Analysis of Variance (PERMANOVA; [35]) considering 3 factors: the fishing Boat (B) as a random factor, the Date (D) as a random factor nested in B, and the trap treatment, fixed and nested in both B and D. Since the two boats operated in different sites, it was not possible to separate the effect of boat and that of site. All analyses were performed with 9999 permutations.

Hatching rates in the field (arcsine-square root transformed) were compared through two-way ANOVA, with the hatching area and the rope material as fixed factors. Mann-Whitney non parametric test was used to detect the rope and hand effects on hatching rates in laboratory.

## Results

### Fishery Data

In 2010, cuttlefish laid eggs on 21 of the 38 ropes, with an average number of eggs of 39.24±44.11 being recorded. No difference in egg numbers was observed between the yellow and blue ropes (Mann-Whitney U-test: Z = 0.25, p = 0.800; N_1_ = 8, N_2_ = 11).

In 2011, 75.5% of the ropes were used for deposition (hemp: 81.6%; elastic: 69.8%). The number of eggs laid on the ropes ranged from 1 to 341 (145.13±114.99), with an estimated density of 7.8±2.94 eggs/cm. No significant difference was found between the two rope materials in the number of ropes with eggs (χ^2^_1_ = 1.34, p = 0.247) or the maximum number of eggs laid (t_41_ = 2.02, p = 0.553).

The number of eggs laid on the traps ranged from 0 to 3077 (947.14±587.03 eggs per trap). Considering the number of traps employed, an average of 3551250±220125 eggs are estimated to likely be destroyed at the end of the breeding season by the 15 fishermen operating in the compartment of Chioggia (2.96 miles long along the coast south of the channel connecting the lagoon to the open sea), corresponding to more than 10000 cuttlefish clutches.

**Figure 4:**
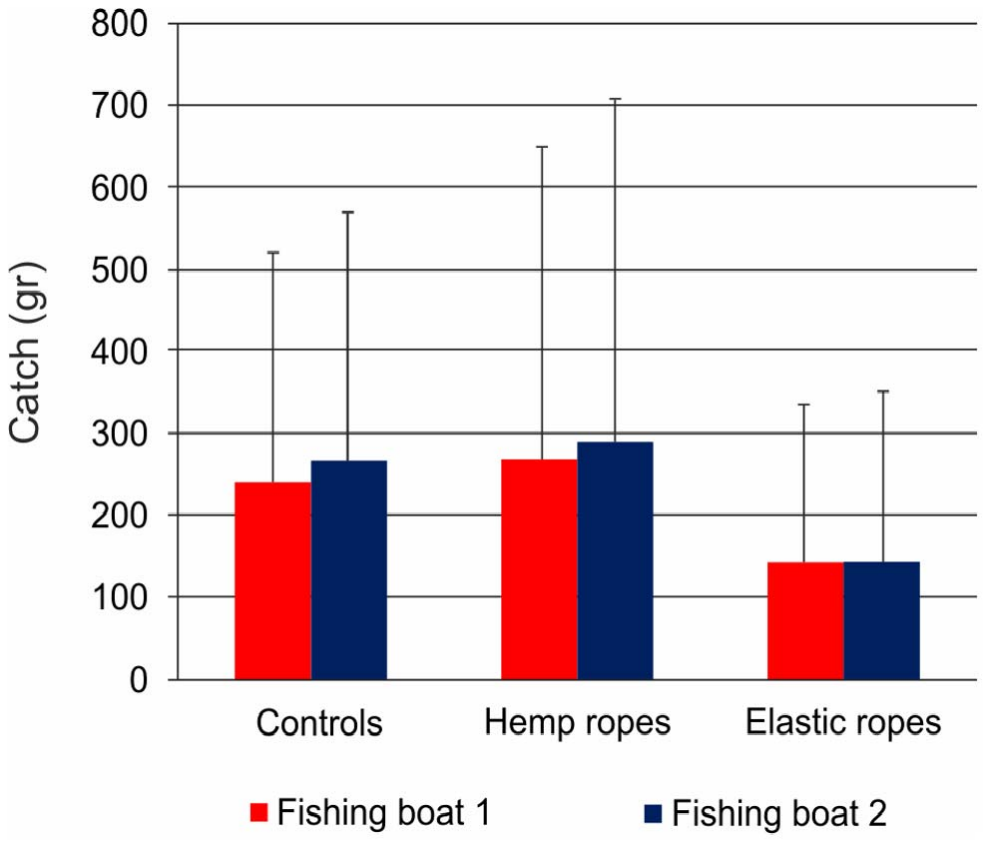
Cuttlefish catches. Average cuttlefish catches (by weight) in traps with hemp ropes, elastic ropes and controls for the two fishing boats. Bars represent standard deviations. The colours are the same as in Figure 1.

The average percentage of eggs on the ropes relative to the total number of eggs laid (rope and trap) was 23.7±24.5%. The number of cuttlefish caught per trap was highly variable (raw data are provided in Table S1), 0.93±1.46 on average, and their weight was 201.04±346.89 g. No significant differences in cuttlefish catches were found between traps with hemp or elastic ropes and the controls or among the fishing boats/sites (Figure 4A), whereas the catch differed among different dates (Table 1), with higher catches recorded at the end of April.

**Table 1:** Results of PERMANOVA for cuttlefish catches in both numbers and weight, based on Euclidean distances, with three factors: Boat, Date and Treatment.

	df	SS	MS	Pseudo-F	P(perm)
**Cuttlefish number**					
Boat	1	0.006	0.006	0.01	0.914
Date(Boat)	12	16.43	1.37	4.95	<0.001
Treatment(Date(Boat))	28	7.74	0.28	0.85	0.690
Residuals	346	113.08	0.33		
Total	387	137.25			
**Cuttlefish weight**					
Boat	1	0.08	0.08	0.003	0.958
Date(Boat)	12	338.32	28.19	4.46	0.001
Treatment(Date(Boat))	28	176.69	6.31	0.77	0.801
Residuals	346	2854.50	8.25		
Total	387	3373.30			

df: degrees of freedom; SS: sum of squares; MS: mean squares; Pseudo-F: pseudo F statistic; P(perm): P permutations.

### Hatching Rates

**Figure 5:**
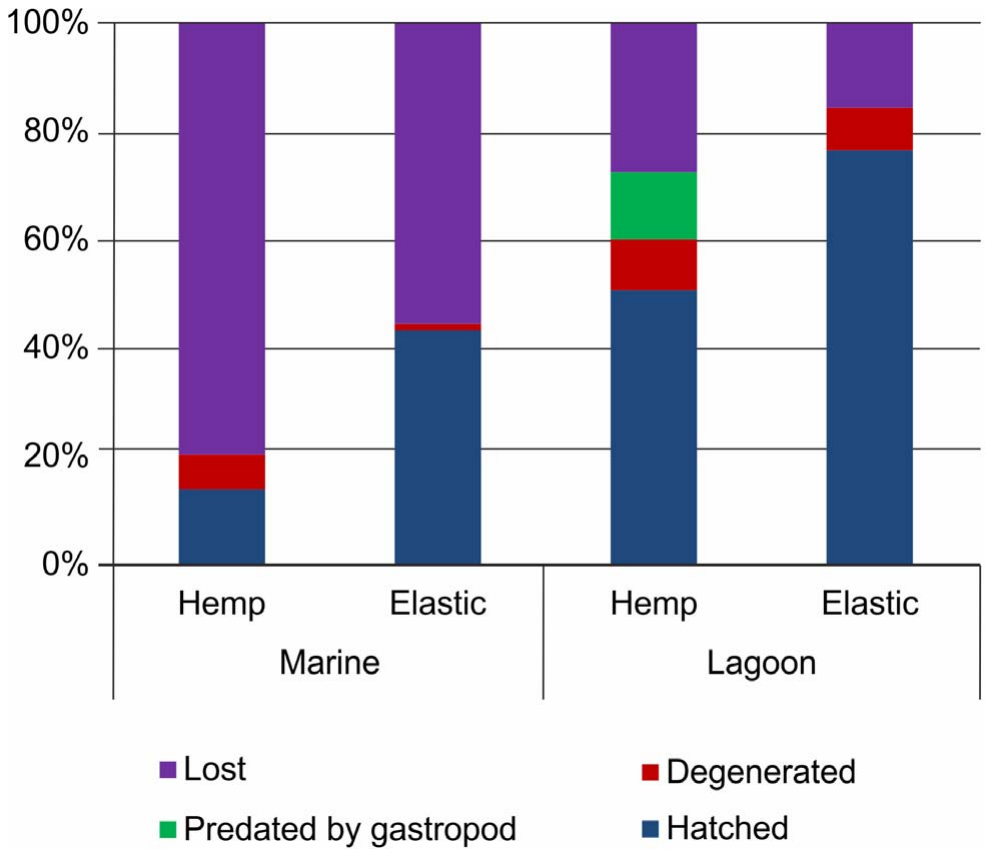
Hatching rates in the field. Percentage of eggs hatched, predated by gastropod, lost and degenerated from hemp and elastic ropes at the marine and lagoon hatching sites.

The percentage of eggs that hatched in the laboratory was 88.0±6.8%, with no difference being detected between ropes (R) and the eggs collected by hand (H) (Mann-Whitney: Z = 1.13, p = 0.256, N_R_ = 9, N_H_ = 13). At the natural sites, the hatching rate was lower than in laboratory (44.7±30.4%). The percentage of eggs that hatched in the lagoon area was significantly higher than in the marine area and was higher on elastic ropes than hemp ones. The percentage of eggs lost, detached from ropes or predated was higher in the marine area and on the hemp ropes. Degeneration was greater in the lagoon, with no significant difference being observed between the two materials. Predation by gastropod was observed only on four ropes in the lagoon site (Figure 5; Table 2; raw data are provided in Table S2). At the hatching sites, the temperature ranged from 17 to 26°C, salinity from 31.15 to 35.79‰, dissolved oxygen from 5.26 to 8.56 mL/L, and pH from 7.85 to 8.37. No differences in the examined parameters were found between areas (Mann-Whitney U-test, for all tests p>0.19).

**Table 2:** Hatching experiments conducted in the field. Results of ANOVA for the percentages of hatched, lost and degenerated eggs, with two factors: Material of rope, Site.

	df	SS	MS	F	p
**Hatched eggs**					
Site	1	1.70	1.70	18.66	<0.001
Material	1	1.12	1.12	12.32	0.001
Site x Material	1	0.02	0.02	0.23	0.638
Residual	30	2.76	0.09		
Total	33	4.85			
**Lost eggs**					
Site	1	2.27	2.27	30.54	<0.001
Material	1	0.39	0.39	5.29	0.029
Site x Material	1	0.02	0.02	0.32	0.578
Error	30	2.23	0.07		
Total	33	4.62			
**Degenerated eggs**					
Site	1	0.14	0.14	4.48	0.043
Material	1	0.04	0.04	1.32	0.260
Site x Material	1	0.05	0.05	1.75	0.196
Error	30	0.93	0.03		
Total	33	1.24			

df: degrees of freedom; SS: sum of squares; MS: mean squares.

### Interviews

**Figure 6:**
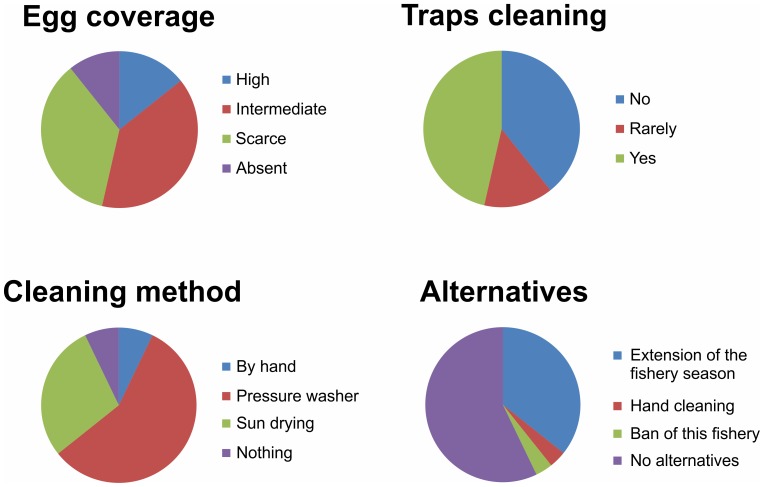
Fisherman interviews. Answers obtained in interviews regarding egg deposition on traps, the occurrence of trap cleaning and trap cleaning procedures, and suggested alternatives to the cleaning procedures.

The majority of the interviewed fishermen reported an intermediate to high egg coverage on their traps during the fishing season (Figure 6). Half of them declared that they cleaned the traps at least once during the season, often through the illegal use of a water-pressure washer in both the middle and the final cleanings of the season. The only alternative method used was the manual removal of eggs, which were then thrown in the water. More than half of the fishermen did not see any possible alternative that would not imply personal economic costs, whereas the others proposed a lengthening of the fishery season to the end of July to allow the hatching of all of the eggs on the traps or banning of this fishery or the manual removal of eggs. A total of 70% of the interviewed fishermen showed positive responses with respect to the proposed mitigation measure.

## Discussion

The results of this study highlighted the high impact of cuttlefish traps on eggs and, through the test of its efficacy and impact on cuttlefish catch in the real-world condition [15], delineate a feasible and effective mitigation measure that could consistently reduce such impacts, directly involving fishermen in the management of this resource.

Traps are used to catch cuttlefish close to the shore in different areas of the Mediterranean Sea, particularly along the western side of the northern-central Adriatic Sea. However, data on the number of traps employed to catch cuttlefish and estimates of the number of eggs laid on traps are not available for other areas, and it is consequently not possible to estimate the overall impact of traps on cuttlefish eggs in the Mediterranean or even in the Adriatic Sea. Nevertheless, even focusing only on the Adriatic Sea, whose western portion includes approximately 350 miles of coast (approximately 120 times the size of the study area), it can be deduced that egg destruction has an important impact on cuttlefish conservation, as reported in the eastern Atlantic [22]. Concern regarding the destruction of eggs laid on traps has also been raised for the trap fisheries of other cuttlefish species, for instance, in the Pacific [23], indicating that the impact of traps on cuttlefish eggs may be common to all fisheries exploiting the breeding behaviour of cuttlefish species.

The mitigation measure proposed in this study is quite effective in reducing the impact of traps on cuttlefish eggs, as can allow more than 20% of eggs to be saved. Considering the difference in the surface available for egg deposition between traps and ropes, this result appears promising and could be improved, for instance, by testing the use of more than two ropes per trap. It is not known where cuttlefish lay eggs in nature in the north-western Adriatic, which is an area characterised by muddy-sandy bottoms, with a shortage of hard substrates. However, cuttlefish are generally known to lay eggs on seagrass [21]. In the study area, seagrass is present only inside the Venetian lagoon, where the seagrass meadows have experienced a marked reduction [36], [37] caused by human activities, including fishing with hydraulic dredges, extensive aquaculture of clams and possibly pollution. This reduction may have contributed to the decline of cuttlefish stocks. The scarcity of natural substrates could encourage the deposition of eggs on artificial substrates, including traps. Indeed, eggs are laid not only on the inner surfaces of traps, but often on the outer ones as well. Moreover, the presence of eggs has been demonstrated to attract mature cuttlefish [38], thereby stimulating egg deposition on traps. The proposed mitigation measure, thanks to the reduction in egg loss experimentally demonstrated in this study, could therefore remarkably contribute to cuttlefish management.

The ropes used in this study did not influence the hatching rate, as demonstrated by the results of laboratory experiments. Moreover, considering the high hatching rates obtained in the laboratory, the manipulation and translocation of ropes from fishing boats to the hatching site (in this case, the laboratory) did not have any substantial effect on hatching rates. The hatching rate in the field was much lower than in the laboratory, which was not attributable to rope manipulations and translocations, as the same procedure was applied in the hatching experiments conducted in the laboratory and in the field. The hatching rates in the field were significantly different between sites and rope materials. The differences in hatching rates between sites are likely related to their different characteristics. The marine area is deeper and exhibits a higher water flow, possibly exposing the ropes to weathering and waves. These characteristics likely caused the higher detachment rate of eggs from ropes in this area in comparison with the lagoon area, which is better sheltered from waves. The higher detachment rate observed on hemp ropes makes them less suitable than elastic ropes for egg development in the field. However, the lagoon area presents shallow water, which may expose the eggs to sudden and unpredictable (although not recorded in this study) variations, for instance, in water temperature, salinity and oxygen concentrations. Sudden variations in abiotic factors and higher proliferation of sessile organisms (V. Melli, personal observation) could have caused the higher degeneration rate observed at this site. In the field hatching experiments, only one infrastructure, one cable and one site per area were employed. The ropes did not come into contact with the cable or the infrastructure, and it is therefore unlikely that these factors could have affected hatching rates, causing the observed differences between sites. However, replicates of sites within marine and lagoon areas are needed to confirm the observed pattern and the role of the different biophysical characteristics of the sites in terms of hatching success. Nevertheless, these results highlighted the need for carefully choosing field hatching sites if this mitigation measure is to be applied and, at the same time, provided initial insight into the selection criteria that should be applied for these sites. The use of elastic ropes in areas characterised by moderate water flow is expected to reduce egg detachment and foster egg development, limiting the proliferation of sessile organisms. Choosing sites characterised by stable abiotic conditions could enhance hatching rates [39], though this point definitely deserves further investigation.

The second aspect that needs to be tested when a mitigation measure is proposed relates to the effects of the measure application on commercial catch [15]. Our results demonstrated that the presence of hemp ropes in the traps did not reduce the catch, and even the lower catch observed in traps with elastic ropes did not differ significantly from the catch of the other examined traps. This finding is extremely relevant for mitigation measure to be accepted. Indeed, fishermen are likely to accept a measure that does not affect their current income more readily than a measure whose positive effects require a time lag to be tangible. Moreover, in the performed interviews the majority of the fishermen declared a positive attitude towards the introduction of the proposed mitigation measure. Therefore, beyond the actual efficacy of reducing egg losses, its lack of impact on cuttlefish eggs and the fisherman attitude towards it make this mitigation measure promising.

Considering our results regarding cuttlefish catches and hatching rates together, a compromise between fisherman and conservation interest can be envisaged. Indeed the use of elastic ropes leads to a lower catch, even if not significant, compared to the use of hemp ropes; however, elastic ropes guaranteed higher hatching rates than hemp ropes at the hatching sites used during this study. Other materials could therefore be tested to maximise hatching rates while maintaining high catches. Moreover, the effect of rope colour on cuttlefish catch was not tested during this study, therefore deserving further investigations.

During the interviews, the fishermen acknowledged the use of destructive methods for trap cleaning, despite the illegality of these methods. Current legislation regarding traps does not propose a feasible and effective alternative to this procedure. Indeed, the manual removal of eggs from traps, which is the only allowed procedure that is actually performed by some fishermen, is not expected to be effective in preserving cuttlefish eggs, as fishermen throw the eggs directly into the water, where they are likely ultimately buried at the bottom, mechanically damaged by contact with the sand, predated or beached. Given that eggs manually removed from traps show a high hatching rate, at least in the laboratory, this procedure could indeed guarantee egg survival if complemented with egg collection and development under controlled conditions; this measure is currently being tested in the central Adriatic Sea (http://www.blumarineservice.it). To reduce egg destruction, some fishermen proposed extension of the fishery season to the end of July, when the majority of eggs should have hatched. This proposal does not represent an effective management strategy because i) it does not prevent egg destruction during the season or when traps are raised and kept on the land, under bad weather conditions (in contrast, ropes could be easily maintained in the water hanged to the boat); ii) it does not ensure the hatching of eggs laid late in the season because egg development lasts from 20 to 50 days [27], which is much longer than the duration of the fishery season; and iii) it would interfere with the hydraulic dredge fishery operating in the area along the coast, usually from the beginning of June.

Other management actions could be developed to limit egg destruction. For instance, a potential alternative management action is the provisioning of fishermen with multiple sets of traps, then collecting those traps with eggs during and at the end of the fishery season and placing them at appropriate sites for egg hatching. However, this measure would also not prevent egg destruction when traps are raised due to bad weather conditions. Moreover, traps are more expensive than ropes. Indeed, the positive attitude of fishermen towards the proposed measure could be related to its inexpensiveness and the fact that it does not alter traditional fishing practices.

To apply the proposed mitigation measure, various steps must be accomplished, their effects tested, and the direct involvement of fishermen is essential. Fishermen are expected to remove ropes from traps when they are covered with eggs or when traps are landed, keep them in buckets with seawater and deliver them to hatching sites. If they are unable to deliver the ropes to the sites the same day they are collected, the ropes could be hung on the boat underwater. Additionally, the hatching sites should be reasonably close to fishery areas, thereby limiting time and costs for fishermen. Hatching sites could exploit structures created for other purposes, such as black mussel farms, which occur along the coast in various areas not only of the Adriatic Sea, but generally in the Mediterranean Sea. Considering the short life cycle of cuttlefish (1–2 years, [40]), positive results and rewards for fishermen in terms of catch are expected to be achieved within a few years and may be monitored using landing data of the fish market of Chioggia, provided both directly by fishermen and by local authorities [29].

## Conclusions

Overall, our results, obtained through integrating experimental data with the knowledge of fishermen highlight the significant impact of trap fishing on cuttlefish and the feasibility of the application of an effective mitigation measure. Cuttlefish are exploited by different types of fisheries and are likely impacted by the reduction of seagrass. Therefore, the management of this species is not easy. However, the mitigation measure proposed and tested here appears to be promising because it i) does not affect the obtained catch and, thus, fishermen’s income, ii) is inexpensive and easy to employ, iii) does not require changes in fishing techniques, iv) generated positive feedback from fishermen and v) would allow the preservation of this traditional artisanal fishery, which is characterised by very low by-catch and impacts on habitats (C. Mazzoldi, unpublished data, [26]). The direct involvement of fishermen in the management of this resource, which is a key premise of this mitigation measure, could make a great contribution to closing the gap between fishery management authorities and stakeholders. The application of this mitigation measure might constitute a first step toward attaining the acknowledgement of a sustainable cuttlefish fishery and, consequently, ecolabeling for cuttlefish caught with traps.

## Supporting Information

Table S1 **Catch of cuttlefish (number and weight) per trap in the three treatments (control, with hemp and elastic ropes).**
(DOCX)

Table S2 **Data from the field hatching experiments of eggs attached to different ropes (hemp and elastic) in the lagoon and marine site.**
(DOCX)
