# Supplementary material for: From Trap to Nursery. Mitigating the Impact of an Artisanal Fishery on Cuttlefish Offspring
Source: PLoS One. 2014 Feb 28;9(2):e90542. doi: 10.1371/journal.pone.0090542 (PMC3938780; doi:10.1371/journal.pone.0090542)
Supplement: Table S1 — Catch of cuttlefish (number and weight) per trap in the three treatments (control, with hemp and elastic ropes). (DOCX) [file pone.0090542.s001.docx]

**Table S1:** Catch of cuttlefish (number and weight) per trap in the three treatments (control, with hemp and elastic ropes.

| **Boat** | **Date** | **Treatment** | **Cuttlefish number** | **Cuttlefish weight (g)** |
| --- | --- | --- | --- | --- |
| Boat 2 | 14/04/2011 | control | 0 | 0 |
| Boat 2 | 14/04/2011 | control | 0 | 0 |
| Boat 2 | 14/04/2011 | control | 1 | 300 |
| Boat 2 | 14/04/2011 | control | 0 | 0 |
| Boat 2 | 14/04/2011 | control | 0 | 0 |
| Boat 2 | 14/04/2011 | control | 2 | 500 |
| Boat 2 | 14/04/2011 | control | 0 | 0 |
| Boat 2 | 14/04/2011 | control | 3 | 650 |
| Boat 2 | 14/04/2011 | control | 0 | 0 |
| Boat 2 | 14/04/2011 | control | 0 | 0 |
| Boat 2 | 14/04/2011 | hemp | 0 | 0 |
| Boat 2 | 14/04/2011 | hemp | 1 | 200 |
| Boat 2 | 14/04/2011 | hemp | 0 | 0 |
| Boat 2 | 14/04/2011 | hemp | 0 | 0 |
| Boat 2 | 14/04/2011 | hemp | 0 | 0 |
| Boat 2 | 14/04/2011 | hemp | 0 | 0 |
| Boat 2 | 14/04/2011 | hemp | 2 | 500 |
| Boat 2 | 14/04/2011 | hemp | 3 | 1100 |
| Boat 2 | 14/04/2011 | hemp | 0 | 0 |
| Boat 2 | 14/04/2011 | hemp | 0 | 0 |
| Boat 2 | 14/04/2011 | elastic | 0 | 0 |
| Boat 2 | 14/04/2011 | elastic | 2 | 800 |
| Boat 2 | 14/04/2011 | elastic | 0 | 0 |
| Boat 2 | 14/04/2011 | elastic | 0 | 0 |
| Boat 2 | 14/04/2011 | elastic | 0 | 0 |
| Boat 2 | 14/04/2011 | elastic | 0 | 0 |
| Boat 2 | 14/04/2011 | elastic | 0 | 0 |
| Boat 2 | 14/04/2011 | elastic | 1 | 200 |
| Boat 2 | 14/04/2011 | elastic | 0 | 0 |
| Boat 2 | 14/04/2011 | elastic | 0 | 0 |
| Boat 1 | 20/04/2011 | control | 0 | 0 |
| Boat 1 | 20/04/2011 | control | 3 | 700 |
| Boat 1 | 20/04/2011 | control | 0 | 0 |
| Boat 1 | 20/04/2011 | control | 3 | 400 |
| Boat 1 | 20/04/2011 | control | 4 | 400 |
| Boat 1 | 20/04/2011 | control | 2 | 700 |
| Boat 1 | 20/04/2011 | control | 0 | 0 |
| Boat 1 | 20/04/2011 | control | 4 | 800 |
| Boat 1 | 20/04/2011 | control | 3 | 700 |
| Boat 1 | 20/04/2011 | control | 2 | 500 |
| Boat 1 | 20/04/2011 | hemp | 0 | 0 |
| Boat 1 | 20/04/2011 | hemp | 0 | 0 |
| Boat 1 | 20/04/2011 | hemp | 5 | 1200 |
| Boat 1 | 20/04/2011 | hemp | 1 | 200 |
| Boat 1 | 20/04/2011 | hemp | 0 | 0 |
| Boat 1 | 20/04/2011 | hemp | 0 | 0 |
| Boat 1 | 20/04/2011 | hemp | 0 | 0 |
| Boat 1 | 20/04/2011 | hemp | 2 | 200 |
| Boat 1 | 20/04/2011 | hemp | 5 | 1200 |
| Boat 1 | 20/04/2011 | hemp | 0 | 0 |
| Boat 1 | 20/04/2011 | elastic | 2 | 400 |
| Boat 1 | 20/04/2011 | elastic | 2 | 600 |
| Boat 1 | 20/04/2011 | elastic | 0 | 0 |
| Boat 1 | 20/04/2011 | elastic | 2 | 400 |
| Boat 1 | 20/04/2011 | elastic | 0 | 0 |
| Boat 1 | 20/04/2011 | elastic | 0 | 0 |
| Boat 1 | 20/04/2011 | elastic | 1 | 300 |
| Boat 1 | 20/04/2011 | elastic | 0 | 0 |
| Boat 1 | 20/04/2011 | elastic | 5 | 1000 |
| Boat 1 | 20/04/2011 | elastic | 2 | 700 |
| Boat 2 | 21/04/2011 | control | 3 | 1000 |
| Boat 2 | 21/04/2011 | control | 3 | 1400 |
| Boat 2 | 21/04/2011 | control | 3 | 500 |
| Boat 2 | 21/04/2011 | control | 0 | 0 |
| Boat 2 | 21/04/2011 | control | 0 | 0 |
| Boat 2 | 21/04/2011 | control | 0 | 0 |
| Boat 2 | 21/04/2011 | control | 4 | 1000 |
| Boat 2 | 21/04/2011 | control | 5 | 1100 |
| Boat 2 | 21/04/2011 | control | 8 | 1600 |
| Boat 2 | 21/04/2011 | control | 1 | 200 |
| Boat 2 | 21/04/2011 | hemp | 2 | 300 |
| Boat 2 | 21/04/2011 | hemp | 4 | 1000 |
| Boat 2 | 21/04/2011 | hemp | 4 | 1100 |
| Boat 2 | 21/04/2011 | hemp | 1 | 300 |
| Boat 2 | 21/04/2011 | hemp | 0 | 0 |
| Boat 2 | 21/04/2011 | hemp | 1 | 300 |
| Boat 2 | 21/04/2011 | hemp | 3 | 900 |
| Boat 2 | 21/04/2011 | hemp | 0 | 0 |
| Boat 2 | 21/04/2011 | hemp | 1 | 200 |
| Boat 2 | 21/04/2011 | hemp | 0 | 0 |
| Boat 2 | 21/04/2011 | elastic | 0 | 0 |
| Boat 2 | 21/04/2011 | elastic | 1 | 200 |
| Boat 2 | 21/04/2011 | elastic | 1 | 500 |
| Boat 2 | 21/04/2011 | elastic | 0 | 0 |
| Boat 2 | 21/04/2011 | elastic | 0 | 0 |
| Boat 2 | 21/04/2011 | elastic | 0 | 0 |
| Boat 2 | 21/04/2011 | elastic | 3 | 800 |
| Boat 2 | 21/04/2011 | elastic | 3 | 600 |
| Boat 2 | 21/04/2011 | elastic | 0 | 0 |
| Boat 2 | 21/04/2011 | elastic | 1 | 200 |
| Boat 1 | 02/05/2011 | control | 0 | 0 |
| Boat 1 | 02/05/2011 | control | 0 | 0 |
| Boat 1 | 02/05/2011 | control | 1 | 100 |
| Boat 1 | 02/05/2011 | control | 2 | 200 |
| Boat 1 | 02/05/2011 | control | 3 | 300 |
| Boat 1 | 02/05/2011 | control | 1 | 100 |
| Boat 1 | 02/05/2011 | control | 5 | 1300 |
| Boat 1 | 02/05/2011 | control | 1 | 100 |
| Boat 1 | 02/05/2011 | control | 2 | 600 |
| Boat 1 | 02/05/2011 | control | 0 | 0 |
| Boat 1 | 02/05/2011 | hemp | 2 | 500 |
| Boat 1 | 02/05/2011 | hemp | 4 | 800 |
| Boat 1 | 02/05/2011 | hemp | 0 | 0 |
| Boat 1 | 02/05/2011 | hemp | 0 | 0 |
| Boat 1 | 02/05/2011 | hemp | 3 | 300 |
| Boat 1 | 02/05/2011 | hemp | 0 | 0 |
| Boat 1 | 02/05/2011 | hemp | 0 | 0 |
| Boat 1 | 02/05/2011 | hemp | 0 | 0 |
| Boat 1 | 02/05/2011 | hemp | 3 | 600 |
| Boat 1 | 02/05/2011 | hemp | 0 | 0 |
| Boat 1 | 02/05/2011 | elastic | 0 | 0 |
| Boat 1 | 02/05/2011 | elastic | 0 | 0 |
| Boat 1 | 02/05/2011 | elastic | 0 | 0 |
| Boat 1 | 02/05/2011 | elastic | 3 | 500 |
| Boat 1 | 02/05/2011 | elastic | 2 | 500 |
| Boat 1 | 02/05/2011 | elastic | 0 | 0 |
| Boat 1 | 02/05/2011 | elastic | 1 | 100 |
| Boat 1 | 02/05/2011 | elastic | 0 | 0 |
| Boat 1 | 02/05/2011 | elastic | 0 | 0 |
| Boat 1 | 02/05/2011 | elastic | 1 | 200 |
| Boat 2 | 06/05/2011 | control | 0 | 0 |
| Boat 2 | 06/05/2011 | control | 2 | 700 |
| Boat 2 | 06/05/2011 | control | 7 | 1900 |
| Boat 2 | 06/05/2011 | control | 1 | 300 |
| Boat 2 | 06/05/2011 | control | 1 | 200 |
| Boat 2 | 06/05/2011 | control | 1 | 280 |
| Boat 2 | 06/05/2011 | control | 1 | 200 |
| Boat 2 | 06/05/2011 | control | 5 | 1100 |
| Boat 2 | 06/05/2011 | control | 1 | 110 |
| Boat 2 | 06/05/2011 | control | 1 | 190 |
| Boat 2 | 06/05/2011 | hemp | 0 | 0 |
| Boat 2 | 06/05/2011 | hemp | 0 | 0 |
| Boat 2 | 06/05/2011 | hemp | 3 | 860 |
| Boat 2 | 06/05/2011 | hemp | 0 | 0 |
| Boat 2 | 06/05/2011 | hemp | 1 | 430 |
| Boat 2 | 06/05/2011 | hemp | 6 | 1000 |
| Boat 2 | 06/05/2011 | hemp | 7 | 1800 |
| Boat 2 | 06/05/2011 | hemp | 0 | 0 |
| Boat 2 | 06/05/2011 | hemp | 0 | 0 |
| Boat 2 | 06/05/2011 | hemp | 7 | 1500 |
| Boat 2 | 06/05/2011 | elastic | 0 | 0 |
| Boat 2 | 06/05/2011 | elastic | 5 | 900 |
| Boat 2 | 06/05/2011 | elastic | 0 | 0 |
| Boat 2 | 06/05/2011 | elastic | 1 | 150 |
| Boat 2 | 06/05/2011 | elastic | 3 | 500 |
| Boat 2 | 06/05/2011 | elastic | 0 | 0 |
| Boat 2 | 06/05/2011 | elastic | 1 | 270 |
| Boat 2 | 06/05/2011 | elastic | 0 | 0 |
| Boat 2 | 06/05/2011 | elastic | 0 | 0 |
| Boat 2 | 06/05/2011 | elastic | 0 | 0 |
| Boat 1 | 12/05/2011 | control | 0 | 0 |
| Boat 1 | 12/05/2011 | control | 0 | 0 |
| Boat 1 | 12/05/2011 | control | 2 | 220 |
| Boat 1 | 12/05/2011 | control | 4 | 620 |
| Boat 1 | 12/05/2011 | control | 1 | 620 |
| Boat 1 | 12/05/2011 | control | 0 | 0 |
| Boat 1 | 12/05/2011 | control | 1 | 120 |
| Boat 1 | 12/05/2011 | control | 2 | 320 |
| Boat 1 | 12/05/2011 | control | 0 | 0 |
| Boat 1 | 12/05/2011 | control | 0 | 0 |
| Boat 1 | 12/05/2011 | hemp | 1 | 170 |
| Boat 1 | 12/05/2011 | hemp | 0 | 0 |
| Boat 1 | 12/05/2011 | hemp | 1 | 120 |
| Boat 1 | 12/05/2011 | hemp | 6 | 1020 |
| Boat 1 | 12/05/2011 | hemp | 4 | 620 |
| Boat 1 | 12/05/2011 | hemp | 0 | 0 |
| Boat 1 | 12/05/2011 | hemp | 0 | 0 |
| Boat 1 | 12/05/2011 | hemp | 3 | 620 |
| Boat 1 | 12/05/2011 | hemp | 5 | 720 |
| Boat 1 | 12/05/2011 | hemp | 0 | 0 |
| Boat 1 | 12/05/2011 | elastic | 0 | 0 |
| Boat 1 | 12/05/2011 | elastic | 2 | 370 |
| Boat 1 | 12/05/2011 | elastic | 2 | 225 |
| Boat 1 | 12/05/2011 | elastic | 2 | 220 |
| Boat 1 | 12/05/2011 | elastic | 0 | 0 |
| Boat 1 | 12/05/2011 | elastic | 0 | 0 |
| Boat 1 | 12/05/2011 | elastic | 0 | 0 |
| Boat 1 | 12/05/2011 | elastic | 1 | 70 |
| Boat 1 | 12/05/2011 | elastic | 3 | 420 |
| Boat 1 | 12/05/2011 | elastic | 0 | 0 |
| Boat 2 | 18/05/2011 | control | 2 | 280 |
| Boat 2 | 18/05/2011 | control | 2 | 390 |
| Boat 2 | 18/05/2011 | control | 1 | 170 |
| Boat 2 | 18/05/2011 | control | 0 | 0 |
| Boat 2 | 18/05/2011 | control | 0 | 0 |
| Boat 2 | 18/05/2011 | control | 0 | 0 |
| Boat 2 | 18/05/2011 | control | 1 | 240 |
| Boat 2 | 18/05/2011 | control | 0 | 0 |
| Boat 2 | 18/05/2011 | control | 0 | 0 |
| Boat 2 | 18/05/2011 | control | 3 | 420 |
| Boat 2 | 18/05/2011 | hemp | 1 | 170 |
| Boat 2 | 18/05/2011 | hemp | 0 | 0 |
| Boat 2 | 18/05/2011 | hemp | 6 | 2220 |
| Boat 2 | 18/05/2011 | hemp | 0 | 0 |
| Boat 2 | 18/05/2011 | hemp | 0 | 0 |
| Boat 2 | 18/05/2011 | hemp | 1 | 280 |
| Boat 2 | 18/05/2011 | hemp | 2 | 420 |
| Boat 2 | 18/05/2011 | hemp | 0 | 0 |
| Boat 2 | 18/05/2011 | hemp | 2 | 280 |
| Boat 2 | 18/05/2011 | hemp | 1 | 150 |
| Boat 2 | 18/05/2011 | elastic | 0 | 0 |
| Boat 2 | 18/05/2011 | elastic | 1 | 190 |
| Boat 2 | 18/05/2011 | elastic | 1 | 170 |
| Boat 2 | 18/05/2011 | elastic | 1 | 150 |
| Boat 2 | 18/05/2011 | elastic | 0 | 0 |
| Boat 2 | 18/05/2011 | elastic | 0 | 0 |
| Boat 2 | 18/05/2011 | elastic | 0 | 0 |
| Boat 2 | 18/05/2011 | elastic | 0 | 0 |
| Boat 2 | 18/05/2011 | elastic | 2 | 350 |
| Boat 2 | 18/05/2011 | elastic | 0 | 0 |
| Boat 1 | 27/05/2011 | control | 0 | 0 |
| Boat 1 | 27/05/2011 | control | 0 | 0 |
| Boat 1 | 27/05/2011 | control | 0 | 0 |
| Boat 1 | 27/05/2011 | control | 0 | 0 |
| Boat 1 | 27/05/2011 | control | 0 | 0 |
| Boat 1 | 27/05/2011 | control | 0 | 0 |
| Boat 1 | 27/05/2011 | control | 0 | 0 |
| Boat 1 | 27/05/2011 | control | 1 | 90 |
| Boat 1 | 27/05/2011 | control | 0 | 0 |
| Boat 1 | 27/05/2011 | control | 1 | 200 |
| Boat 1 | 27/05/2011 | hemp | 2 | 200 |
| Boat 1 | 27/05/2011 | hemp | 0 | 0 |
| Boat 1 | 27/05/2011 | hemp | 0 | 0 |
| Boat 1 | 27/05/2011 | hemp | 0 | 0 |
| Boat 1 | 27/05/2011 | hemp | 0 | 0 |
| Boat 1 | 27/05/2011 | hemp | 1 | 120 |
| Boat 1 | 27/05/2011 | hemp | 0 | 0 |
| Boat 1 | 27/05/2011 | hemp | 4 | 520 |
| Boat 1 | 27/05/2011 | hemp | 2 | 300 |
| Boat 1 | 27/05/2011 | hemp | 2 | 340 |
| Boat 1 | 27/05/2011 | elastic | 0 | 0 |
| Boat 1 | 27/05/2011 | elastic | 0 | 0 |
| Boat 1 | 27/05/2011 | elastic | 1 | 170 |
| Boat 1 | 27/05/2011 | elastic | 0 | 0 |
| Boat 1 | 27/05/2011 | elastic | 0 | 0 |
| Boat 1 | 27/05/2011 | elastic | 1 | 50 |
| Boat 1 | 27/05/2011 | elastic | 3 | 300 |
| Boat 1 | 27/05/2011 | elastic | 0 | 0 |
| Boat 1 | 27/05/2011 | elastic | 0 | 0 |
| Boat 1 | 27/05/2011 | elastic | 1 | 250 |
| Boat 2 | 27/05/2011 | control | 0 | 0 |
| Boat 2 | 27/05/2011 | control | 0 | 0 |
| Boat 2 | 27/05/2011 | control | 0 | 0 |
| Boat 2 | 27/05/2011 | control | 0 | 0 |
| Boat 2 | 27/05/2011 | control | 0 | 0 |
| Boat 2 | 27/05/2011 | control | 0 | 0 |
| Boat 2 | 27/05/2011 | control | 0 | 0 |
| Boat 2 | 27/05/2011 | control | 0 | 0 |
| Boat 2 | 27/05/2011 | control | 1 | 210 |
| Boat 2 | 27/05/2011 | control | 0 | 0 |
| Boat 2 | 27/05/2011 | hemp | 0 | 0 |
| Boat 2 | 27/05/2011 | hemp | 2 | 340 |
| Boat 2 | 27/05/2011 | hemp | 0 | 0 |
| Boat 2 | 27/05/2011 | hemp | 2 | 220 |
| Boat 2 | 27/05/2011 | hemp | 0 | 0 |
| Boat 2 | 27/05/2011 | hemp | 1 | 760 |
| Boat 2 | 27/05/2011 | hemp | 0 | 0 |
| Boat 2 | 27/05/2011 | hemp | 0 | 0 |
| Boat 2 | 27/05/2011 | hemp | 0 | 0 |
| Boat 2 | 27/05/2011 | hemp | 0 | 0 |
| Boat 2 | 27/05/2011 | elastic | 0 | 0 |
| Boat 2 | 27/05/2011 | elastic | 1 | 200 |
| Boat 2 | 27/05/2011 | elastic | 0 | 0 |
| Boat 2 | 27/05/2011 | elastic | 0 | 0 |
| Boat 2 | 27/05/2011 | elastic | 0 | 0 |
| Boat 2 | 27/05/2011 | elastic | 0 | 0 |
| Boat 2 | 27/05/2011 | elastic | 0 | 0 |
| Boat 2 | 27/05/2011 | elastic | 0 | 0 |
| Boat 2 | 27/05/2011 | elastic | 0 | 0 |
| Boat 2 | 27/05/2011 | elastic | 0 | 0 |
| Boat 1 | 10/06/2011 | control | 0 | 0 |
| Boat 1 | 10/06/2011 | control | 0 | 0 |
| Boat 1 | 10/06/2011 | control | 2 | 320 |
| Boat 1 | 10/06/2011 | control | 0 | 0 |
| Boat 1 | 10/06/2011 | control | 0 | 0 |
| Boat 1 | 10/06/2011 | control | 0 | 0 |
| Boat 1 | 10/06/2011 | control | 0 | 0 |
| Boat 1 | 10/06/2011 | control | 0 | 0 |
| Boat 1 | 10/06/2011 | control | 0 | 0 |
| Boat 1 | 10/06/2011 | control | 0 | 0 |
| Boat 1 | 10/06/2011 | hemp | 0 | 0 |
| Boat 1 | 10/06/2011 | hemp | 1 | 70 |
| Boat 1 | 10/06/2011 | hemp | 0 | 0 |
| Boat 1 | 10/06/2011 | hemp | 0 | 0 |
| Boat 1 | 10/06/2011 | hemp | 3 | 380 |
| Boat 1 | 10/06/2011 | hemp | 0 | 0 |
| Boat 1 | 10/06/2011 | hemp | 3 | 410 |
| Boat 1 | 10/06/2011 | hemp | 0 | 0 |
| Boat 1 | 10/06/2011 | hemp | 0 | 0 |
| Boat 1 | 10/06/2011 | elastic | 0 | 0 |
| Boat 1 | 10/06/2011 | elastic | 1 | 120 |
| Boat 1 | 10/06/2011 | elastic | 2 | 350 |
| Boat 1 | 10/06/2011 | elastic | 0 | 0 |
| Boat 1 | 10/06/2011 | elastic | 0 | 0 |
| Boat 1 | 10/06/2011 | elastic | 1 | 110 |
| Boat 1 | 10/06/2011 | elastic | 2 | 360 |
| Boat 1 | 10/06/2011 | elastic | 0 | 0 |
| Boat 1 | 17/06/2011 | control | 0 | 0 |
| Boat 1 | 17/06/2011 | control | 2 | 310 |
| Boat 1 | 17/06/2011 | control | 1 | 150 |
| Boat 1 | 17/06/2011 | control | 3 | 700 |
| Boat 1 | 17/06/2011 | control | 0 | 0 |
| Boat 1 | 17/06/2011 | control | 0 | 0 |
| Boat 1 | 17/06/2011 | control | 0 | 0 |
| Boat 1 | 17/06/2011 | control | 0 | 0 |
| Boat 1 | 17/06/2011 | control | 0 | 0 |
| Boat 1 | 17/06/2011 | control | 0 | 0 |
| Boat 1 | 17/06/2011 | hemp | 0 | 0 |
| Boat 1 | 17/06/2011 | hemp | 0 | 0 |
| Boat 1 | 17/06/2011 | hemp | 1 | 230 |
| Boat 1 | 17/06/2011 | hemp | 1 | 180 |
| Boat 1 | 17/06/2011 | hemp | 0 | 0 |
| Boat 1 | 17/06/2011 | hemp | 0 | 0 |
| Boat 1 | 17/06/2011 | hemp | 2 | 610 |
| Boat 1 | 17/06/2011 | hemp | 2 | 440 |
| Boat 1 | 17/06/2011 | hemp | 0 | 0 |
| Boat 1 | 17/06/2011 | elastic | 0 | 0 |
| Boat 1 | 17/06/2011 | elastic | 2 | 370 |
| Boat 1 | 17/06/2011 | elastic | 0 | 0 |
| Boat 1 | 17/06/2011 | elastic | 0 | 0 |
| Boat 1 | 17/06/2011 | elastic | 2 | 280 |
| Boat 1 | 17/06/2011 | elastic | 1 | 200 |
| Boat 1 | 17/06/2011 | elastic | 0 | 0 |
| Boat 1 | 17/06/2011 | elastic | 2 | 250 |
| Boat 1 | 17/06/2011 | elastic | 1 | 100 |
| Boat 1 | 17/06/2011 | elastic | 0 | 0 |
