# Supplementary material for: From Trap to Nursery. Mitigating the Impact of an Artisanal Fishery on Cuttlefish Offspring
Source: PLoS One. 2014 Feb 28;9(2):e90542. doi: 10.1371/journal.pone.0090542 (PMC3938780; doi:10.1371/journal.pone.0090542)
Supplement: Table S2 — Data from the field hatching experiments of eggs attached to different ropes (hemp and elastic) in the lagoon and marine site. (DOCX) [file pone.0090542.s002.docx]

**Table S2:** Data from the field hatching experiments of eggs attached to different ropes (hemp and elastic) in the lagoon and marine site.

| **Site** | **Rope ID** | **Rope material** | **Date of deployment** | **Total egg number** | **Hatched eggs** | **Eggs predated by gastropds** | **Degenerated eggs** | **Lost eggs** |
| --- | --- | --- | --- | --- | --- | --- | --- | --- |
| Lagoon | 3 | elastic | 13/06/2011 | 122 | 54 | 0 | 11 | 57 |
| Marine | 6 | elastic | 13/06/2011 | 59 | 36 | 0 | 0 | 23 |
| Marine | 13 | elastic | 10/05/2011 | 312 | 100 | 0 | 0 | 212 |
| Marine | 14 | elastic | 10/05/2011 | 135 | 61 | 0 | 0 | 74 |
| Marine | 18 | elastic | 10/05/2011 | 200 | 54 | 0 | 0 | 146 |
| Lagoon | 26 | hemp | 09/05/2011 | 221 | 106 | 13 | 0 | 102 |
| Marine | 29 | hemp | 10/05/2011 | 87 | 0 | 0 | 14 | 73 |
| Lagoon | 32 | hemp | 09/05/2011 | 337 | 113 | 147 | 0 | 77 |
| Marine | 35 | hemp | 10/05/2011 | 349 | 51 | 0 | 19 | 279 |
| Marine | 43 | elastic | 20/06/2011 | 123 | 35 | 0 | 20 | 68 |
| Marine | 44 | elastic | 20/06/2011 | 78 | 4 | 0 | 0 | 74 |
| Lagoon | 46 | elastic | 09/05/2011 | 123 | 102 | 0 | 11 | 10 |
| Marine | 51 | elastic | 20/06/2011 | 29 | 4 | 0 | 0 | 25 |
| Marine | 52 | elastic | 20/06/2011 | 33 | 17 | 0 | 0 | 16 |
| Lagoon | 54 | elastic | 20/06/2011 | 73 | 54 | 0 | 16 | 3 |
| Marine | 55 | elastic | 10/05/2011 | 51 | 15 | 0 | 0 | 36 |
| Marine | 57 | elastic | 10/05/2011 | 79 | 70 | 0 | 0 | 9 |
| Marine | 58 | elastic | 18/05/2011 | 148 | 122 | 0 | 0 | 26 |
| Lagoon | 63 | hemp | 09/05/2011 | 272 | 88 | 118 | 7 | 59 |
| Lagoon | 64 | hemp | 09/05/2011 | 204 | 138 | 8 | 18 | 40 |
| Marine | 66 | hemp | 20/06/2011 | 153 | 57 | 0 | 0 | 96 |
| Lagoon | 67 | hemp | 09/05/2011 | 187 | 159 | 4 | 7 | 17 |
| Marine | 68 | hemp | 20/06/2011 | 129 | 53 | 0 | 0 | 76 |
| Marine | 75 | hemp | 20/06/2011 | 79 | 3 | 0 | 0 | 76 |
| Lagoon | 77 | hemp | 18/05/2011 | 248 | 176 | 0 | 22 | 50 |
| Lagoon | 78 | hemp | 13/06/2011 | 214 | 55 | 0 | 6 | 153 |
| Lagoon | 81 | elastic | 18/05/2011 | 112 | 103 | 0 | 3 | 6 |
| Marine | 82 | elastic | 18/05/2011 | 133 | 119 | 0 | 0 | 14 |
| Marine | 95 | hemp | 13/06/2011 | 168 | 0 | 0 | 29 | 139 |
| Marine | 103 | hemp | 13/06/2011 | 109 | 0 | 0 | 6 | 103 |
| Lagoon | 109 | hemp | 20/06/2011 | 41 | 17 | 0 | 20 | 4 |
| Marine | 112 | elastic | 20/06/2011 | 188 | 13 | 0 | 0 | 175 |
| Lagoon | A | elastic | 11/07/2011 | 188 | 194* | 0 | 0 | 6 |
| Lagoon | B | elastic | 11/07/2011 | 38 | 26 | 0 | 2 | 10 |

*A total of twelve new eggs were laid on this rope during the hatching experiment.
